# Modified Broadband Ruthroff-Type Transmission Line Transformer Balun for Isolation-Enhanced Passive Mixer Design

**DOI:** 10.3390/mi15030332

**Published:** 2024-02-28

**Authors:** Ding He, Zhentao Yu, Jie Chen, Kaiyuan Du, Zhiqiang Zhu, Pu Cheng, Cheng Tan

**Affiliations:** 1 Aerospace Information Research Institute, Chinese Academy of Sciences, Beijing 100190, China; heding201@mails.ucas.ac.cn (D.H.);; 2 School of Electronic, Electrical and Communication Engineering, University of Chinese Academy of Sciences, Beijing 101408, China; 3 Naval Submarine Academy, Qingdao 266000, China; 4 Institute of Information Engineering, Chinese Academy of Sciences, Beijing 100085, China

**Keywords:** compensation network, double balanced mixer (DBM), GaAs, isolation enhancement, Ruthroff-type balun, transmission line transformer (TLT)

## Abstract

Generalized broadband operation facilitates multifunction or multiband highly integrated applications, such as modern transceiver systems, where ultra-wideband bidirectional passive mixers are favored to avoid a complex up/down-conversion scheme. In this paper, a modified Ruthroff-type transmission line transformer (TLT) balun is presented to enhance the isolation of the mixer from the local oscillator (LO) to the radio frequency (RF). Compared to the conventional methods, the proposed Ruthroff-type architecture adopts a combination of shunt capacitors and parallel coupled lines to improve the return loss at the LO port, thus effectively avoiding the area consumption for the diode-to-balun impedance transformation while simultaneously providing a suitable point for IF extraction. In addition, a parallel compensation technique consisting of an inductor and resistor is applied to the RF balun to significantly improve the amplitude/phase balance performance over a wide bandwidth. Benefiting from the aforementioned operations, an isolation-enhanced 8–30 GHz passive double-balanced mixer is designed as a proof-of-principle demonstration via 0.15-micrometer GaAs p-HEMT technology. It exhibits ultra-broadband performance with 7 dB average conversion loss and 50 dB LO-to-RF isolation under 15 dBm LO power. The monolithic microwave integrated circuit area is 0.96 × 1.68 mm^2^ including all pads.

## 1. Introduction

With the ongoing integration and multifunctionality of modern wireless transceivers, ultra-wideband multiplexable module design is recognized as a promising means of producing low-cost, highly reliable compact systems [1,2,3,4]. As one of the essential components of RF communication front-ends, high-performance mixers are widely demanded to economically and efficiently operationalize the up/down spectrum shifting functions of signals. There are various circuit topologies and electrical configurations for mixers, which are commonly categorized into two groups, the active type [5,6,7,8,9] and passive type [10,11,12,13,14,15,16,17,18,19,20,21,22], further classified as single-ended, single-balanced, double-balanced, triple-balanced, and subharmonic approaches. In addition, the specific selection criteria should be determined based on system specifications, including operating bandwidth, conversion loss, port isolation, and compactness [23]. In comparison to the other aforementioned architectures, GaAs-based passive double-balanced mixers (DBMs) are capable of performing spectral shifting functions over a wider bandwidth range. In addition, DBMs are favored due to several major advantages and relatively compact area [24,25]: (1) the theoretically perfect suppression of even harmonics of the LO and RF signals, resulting in a pure spectrum; (2) high isolation performance within a broadband range without the insertion of filtering circuits, which brings great convenience to the application; and (3) no dc power dissipation and the IF extraction method is dc-coupled.

**Figure 1:**
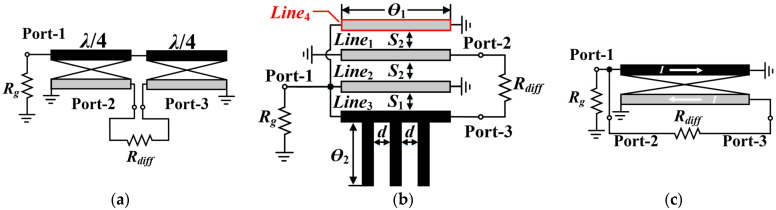
Schematics of transmission line baluns. (**a**) Marchand balun; (**b**) MCL balun; (**c**) Ruthroff-type balun.

For passive DBM design, the RF performance of the balun is the main contributing factor to the bandwidth of the mixer. Especially regarding the isolation metric, a theoretical balun with perfectly differentiated output signals would make the mixer’s isolation infinite. Therefore, numerous studies so far have focused on the wideband baluns, the most common of which is the Marchand balun with a theoretically ideal 180° phase difference, as illustrated in Figure 1a. However, practically, the amplitude/phase characteristics and insertion loss (IL) of the Marchand balun will deteriorate in the high-frequency and broadband case. Additionally, it consists of two sets of λ/4 coupled lines and thus consumes more area. Study [16] proposed a novel multi-conductor coupled-line (MCL) balun (shown in Figure 1b), which can achieve superior amplitude/phase balance performance and effectively reduce the balun size. However, the periodic open stubs employed in the artificial transmission line are unfavorable for layouts in low-frequency situations due to the inability to be folded. Apart from the two aforementioned types, the Ruthroff-type balun, shown in Figure 1c, is another good choice for optimizing compactness and RF performance. Its physical length is generally less than 1/8 λ, which makes it feasible to be more compact. In addition, a Ruthroff balun ideally prevents resonance and shows greater bandwidth and lower IL since stray inductance and capacitance will be absorbed into the characteristic impedance of the transmission line. Nevertheless, such bifilar wound transmission line transformer (TLT) baluns still suffer from the drawback that the impedance transformation ratio of the unbalanced port to the balanced port is fixed at 1:4, which limits its application in impedance step-down situations. In addition, there are few Ruthroff balun designs implemented via standard semiconductor technologies since bifilar wound TLT baluns typically require ferrite materials.

In this article, we propose an on-chip modified Ruthroff-type TLT balun for the implementation of passive DBM. The isolation between LO and RF is enhanced due to the superior broadband amplitude–phase characteristics of the presented balun. The major contributions are as follows: (1) The improved Ruthroff balun based on edge-coupled spirals is deployed at the LO port. Compared to traditional complex matching schemes, impedance transformation from the unbalanced ports to the diodes can be simply achieved by using a shunt capacitor and U section located across the unbalanced ports, thus facilitating the LO standing wave optimization. Moreover, the U section also provides a suitable IF extraction point and introduces other advantages, including additional even-mode rejection and improved balancing performance. (2) At the RF port, a parallel compensation network consisting of a resistor and inductor is adopted to improve the balanced performance of the Ruthroff balun. The compensation mechanism is analyzed in detail from the view of the inversed parallel coupled line. Based on the above-mentioned techniques, 8–30 GHz was designed for demonstration via 0.15 µm GaAs p-HEMT technology.

Section 2 elucidates the adopted GaAs process. Section 3 illustrates the concept and challenges of the TLT baluns. Improvements for step-down conversion are proposed. Moreover, compensation techniques used to optimize the amplitude–phase characteristics are also introduced and analyzed in detail. Section 4 presents the performance results of the DBM designed from two proposed baluns. Finally, Section 5 provides the conclusion.

## 2. The Adopted GaAs Technology

**Figure 2:**
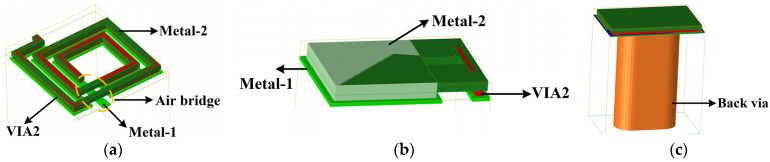
Three-dimensional models of (**a**) inductor; (**b**) MIM capacitor; and (**c**) GND.

In this paper, the proposed design is based on WIN Semiconductor Corp’s commercial 0.15 µm GaAs p-HEMT process (i.e., PL1512), which consists of a substrate of GaAs with a thickness of 100 µm (dielectric constant of 12.9), and air–dielectric and SiN–dielectric layers with thicknesses of 0.15 µm and 2.3 µm, respectively (dielectric constant of 6.9). The technology provides two metal layers, which from the top to the bottom are Metal-2 and Metal-1, with thicknesses of 4 µm and 1.33 µm, respectively. By using the via holes and bimetallic layers, a wide range of passive components can be fabricated such as thin film resistors (TFRs) with a square resistance of 50 ohms, inductors, filters, TLTs, baluns, metal–insulator–metal (MIM) capacitors, and power dividers. As immediately seen in Figure 2a, Metal-1 and Metal-2 are wound to form a planar inductor by connecting through VIA2, and its other end outputs a signal through Metal-1 under several air bridges. Figure 2b shows the structure of the MIM capacitor, which consists of Metal-1 and Metal-2 forming a parallel plate capacitor, which in turn can be tuned by adjusting the size of the metal area to obtain a suitable value. The GND pads are made of bimetal connected to the bottom cover through back via holes as shown in Figure 2c. In addition, it offers diode models for millimeter-wave bands. Since the process features two advantages, (1) the thick metal layer reduces the conductive loss and (2) the high-resistance substrate helps to block the substrate loss, the process drastically improves the quality factor of passive components, which effectively reduces the dielectric loss and conduction loss. As a result, the technology is ideally suited for monolithic microwave-integrated circuit designs with mixers, attenuators, and phase shifters.

## 3. Analysis of and Improvement in TLT Balun

### 3.1. Concept and Challenges of Conventional Ruthroff-Type TLT Balun

**Figure 3:**
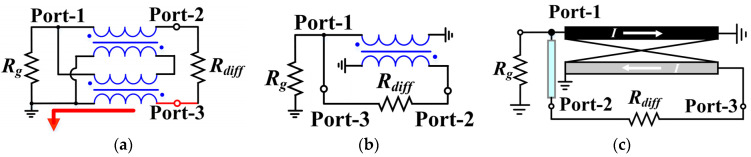
Schematics of bifilar wound TLT baluns. (**a**) Guanella type; (**b**) Ruthroff type; (**c**) Ruthroff-type balun with compensation line.

TLTs are commonly applied from low frequencies to UHF [26], which are typically served as impedance transformers with high power capacity and low insertion loss. Figure 2a,b show the most popular TLT architectures, i.e., Guanella-type and Ruthroff-type designs [27], respectively. Although the former has a flexible step-down impedance transformation ratio, it severely relies on ferrite material to enhance the inductance. For planar circuits, insufficient inductance results in a poor RF choke from Port-3 to the ground and thus significantly deteriorates the amplitude–phase characteristics, as illustrated by the red-highlighted path in Figure 3a. Consequently, the Guanella-type design is not suitable for semiconductor process implementation. Contrarily, the Ruthroff-type balun has a relatively more symmetrical RF-to-ground loop at the output balanced ports, and hence exhibits better amplitude–phase balance performance in planar circuits without ferrite material. In addition, it consists of only one pair of coupled transmission lines, which leads to a more compact layout. 

**Figure 4:**
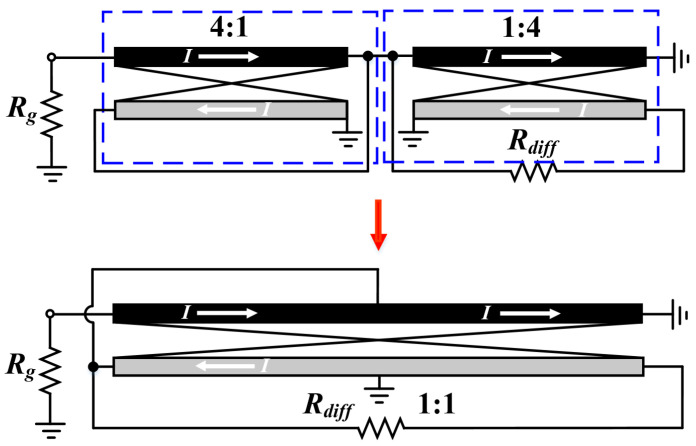
Ruthroff-type balun with impedance transformation of 1:1 proposed in [28].

The above analysis indicates that the Ruthroff-type balun is more appropriate to be applied in semiconductor technology for applications with large bandwidths and reduced dimensions. Nevertheless, there are two concerns that need to be addressed: (1) inadequate phase balanced performance due to phase delays in the coupled lines, and (2) a fixed 1:4 step-up conversion ratio, which severely limits its applicability in RF circuits, especially in inverted class-F differential amplifier and passive balanced mixer designs (which require a step-down impedance transformation). With respect to aforementioned issue-1, it can be alleviated by adding a phase-compensated transmission line from the unbalanced port to the balanced port, as shown in Figure 3c. However, there is as yet no highly satisfactory solution to issue-2. Study [28] suggests a novel step-down conversion Ruthroff balun, which can be regarded as a “back-to-back” combination of two step-up baluns, as schematically illustrated in Figure 4 (1:1 conversion ratio is taken as an example here). The desired conversion ratio can be obtained by adjusting the coupled-line parameters, yet at the cost of a complicated network and larger area consumption, which is not acceptable for a low cost. Therefore, it is a challenge to acquire a compact on-chip broadband Ruthroff balun with a flexible step-down transformation ratio.

### 3.2. Modified LO Ruthroff Balun with Shunt Capacitances and U Section

**Figure 5:**
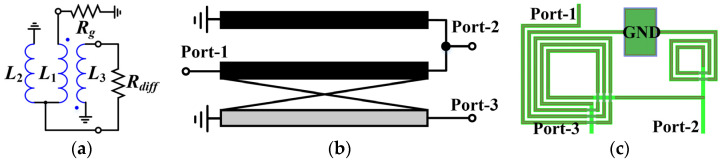
Improved Ruthroff-type TLT balun: (**a**) equivalent circuit; (**b**) schematic; (**c**) layout.

In this article, an improved Ruthroff-type TLT balun is proposed at the LO port, which consists of a core balun, shunt capacitors, and a U section. The equivalent circuit of the adopted core Ruthroff balun is shown in Figure 5a, which utilizes a triple inductor configuration wound by transmission line spirals. The corresponding schematic and layout are shown in Figure 5b,c, respectively. Port-1 is for feeding an RF single-ended signal, where inductors *L*_1_ and *L*_3_ are wound in parallel and negatively coupled. Port-2 is the pass-through end, while Port-3 is the coupled end for producing a signal that is inverted from the pass-through end, and together they form a differential output. Compared to the phase compensation line in Figure 3c, the *L*_2_ of this design affects not only the phase imbalance in the full frequency band, but also the amplitude imbalance since the signal magnitude at Port-2 is dependent on the voltage division of the *L*_2_.

**Figure 6:**
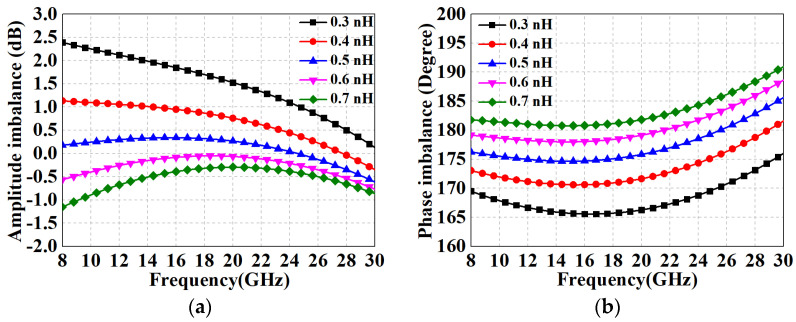
Simulated results with various ideal *L*_2_: (**a**) amplitude imbalance; (**b**) phase imbalance.

**Figure 7:**
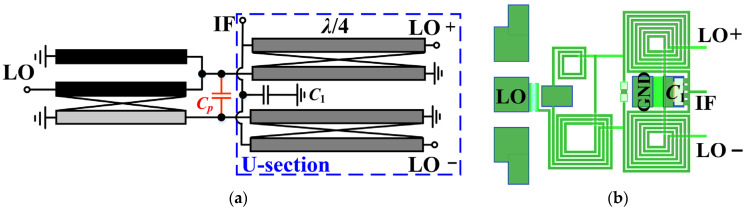
Complete improved LO balun: (**a**) schematic; (**b**) layout.

Figure 6 illustrates the simulation results of the amplitude–phase characteristics of the modified core balun in Figure 5c for different ideal *L*_2_. It can be observed that the inductance *L*_2_ takes values between 0.5 nH and 0.6 nH to have the finest performance. After acquiring the initial value, optimized tuning based on subsequent cascade simulations is still required. The above data indicate that this three-line balun has excellent broadband capability. However, in practice, due to the low input impedance of the diodes, a step-down impedance transformation is necessary prior to use. To address this problem, this paper proposes a solution with a combination of shunt capacitors *C_p_* and U section coupled lines (in blue dashed box), whose complete circuit is shown in Figure 7. As can be seen in Figure 7b, the shunt capacitances are symmetrically distributed between the balanced output ports of the core balun, while the network consisting of two additional sets of parallel coupled lines is referred to as the U section due to its shape. In this case, the U section can be approximated as a distributed network composed of inductors and capacitors, which together with the *C_p_* form a “lumped-distributed” hybrid *LC*-ladder network for step-down impedance conversion. Compared with the “back-to-back” structure in Figure 4, the proposed method features two advantages: (1) only one core balun is required to avoid the complex transformation process, thus significantly economizing the area, and (2) in fact, one end of the secondary coil in Figure 4 still needs an extra microstrip line to connect to the midpoint of the main line, which not only affects the amplitude–phase performance but is also not conducive to the layout, whereas the proposed solution circumvents the use of connecting lines as much as possible.

**Figure 8:**
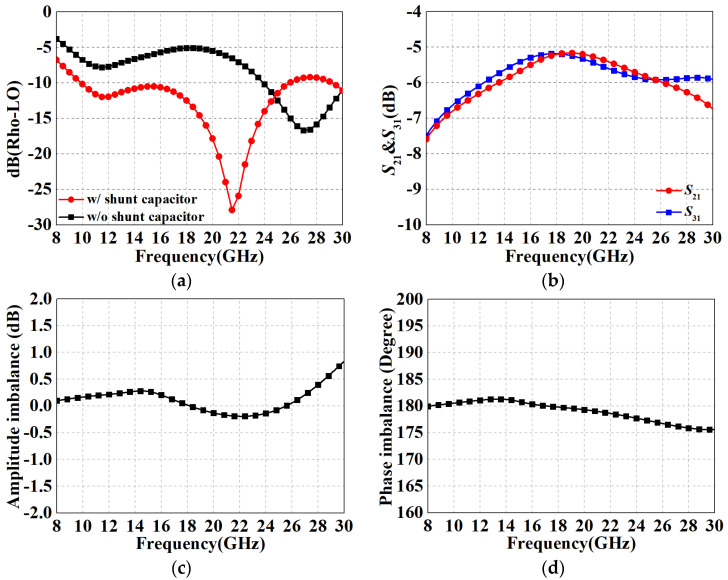
Performance results of the proposed complete LO Ruthroff-type balun circuit. (**a**) Return loss of LO port with/without shunt capacitor; (**b**) *S*_21_ and *S*_31_; (**c**) amplitude imbalance; (**d**) phase imbalance.

In addition, the U section has other effects: (1) It provides additional even-mode suppression, which improves the balance of the balun, thereby increasing port isolation and rejection of the even-order spurious response of the mixer. (2) Since the outputs of the individual core balun have only a virtual ground center and are sensitive to the port impedance, the U section provides a convenient ground return for IF currents and does not deteriorate the balun performance through impedance transformation. Figure 8a shows the return loss of the LO port before and after adding the shunt capacitor, demonstrating that *C_p_* can effectively improve the impedance matching performance. Figure 8b illustrates the insertion loss of the two differential paths for the structure of Figure 7b, with in-band averages of −6.25 dB. Moreover, according to the results in Figure 8c,d, the amplitude/phase imbalance is less than 0.8 dB and 4.5°, respectively, in the range of 8–30 GHz, which reveal a favorable broadband characteristic. Table 1 summarizes the properties with comparison to simulated and measured results of previous works, and it can be observed that the overall performance of the proposed modified Ruthroff-type TLT balun is at the above-intermediate level. The highlight of this work is the ability to maintain superior amplitude/phase balance characteristics over a relatively compact area and wide operating range, even exceeding CMOS process designs for some RF performances.

### 3.3. Modified RF Balun with Parallel Compensation Networks

**Figure 9:**
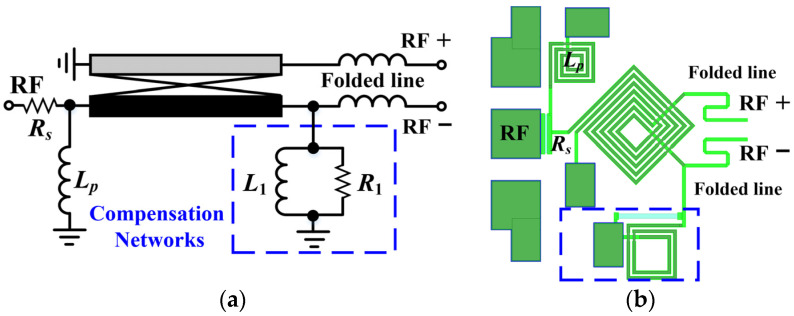
Improved RF balun with compensation network: (**a**) schematic; (**b**) layout.

Since the IF extraction point is positioned at the LO balun, the architecture of the RF balun can be designed to be simpler, with the corresponding schematic and layout illustrated in Figure 9a,b, respectively. With the single parallel coupled line balun, it is hard to ensure favorable amplitude and phase characteristics in broadband applications, which is the major reason why passive DBMs mostly use Marchand baluns instead of edge-coupled line baluns. In this paper, based on the reverse shorted coupled line structure, better amplitude/phase characteristics can be achieved by adding a compensation network consisting of resistor *R*_1_ and inductor *L*_1_. Actually, this can be considered as a modified version of the Ruthroff TLT balun. As can be observed in Figure 9b, to mitigate the deterioration of return loss due to the low input impedance of the diodes, additional matching components are introduced including series resistor *R_s_*, shunt inductor *L_p_*, and series folded lines. Additionally, it is worth mentioning that the introduction of series resistance also contributes to enhance the 1 dB power compression point, although it leads to a slight increase in conversion loss of the DBM.

**Figure 10:**
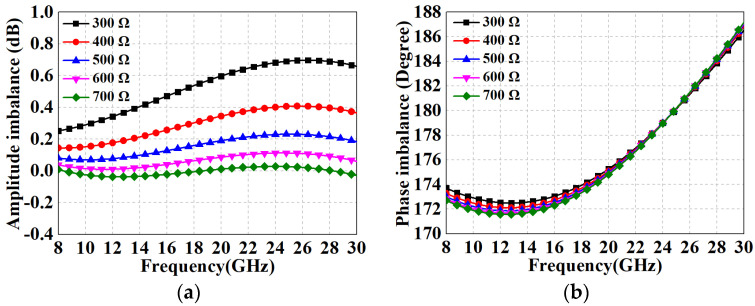
Simulation results of RF balun with various *R*_1_ (where *L*_1_ is fixed at 1 nH): (**a**) amplitude imbalance; (**b**) phase imbalance.

**Figure 11:**
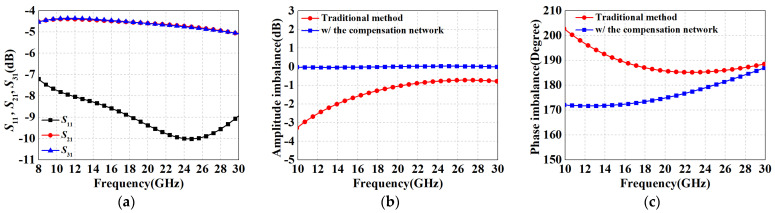
Performance results of the proposed RF Ruthroff-type balun. (**a**) *S*_11_, *S*_21_, and *S*_31_; (**b**) amplitude imbalance; (**c**) phase imbalance.

Figure 10 demonstrates the amplitude–phase imbalance simulation results for the proposed RF balun with different resistances when the fixed compensation inductance is 1 nH. It can be seen that the resistor has a more pronounced effect on the amplitude error regulation while it hardly affects the phase imbalance; on the contrary, *L*_1_ is the main mechanism influencing the phase error. According to the electromagnetic (EM) simulation results, the optimal range of *R*_1_ should be taken near 600 Ω. After several iterations of tuning, the performance of the resulting RF balun is shown in Figure 11. In the range of 8-30 GHz, the IL curves of the two paths nearly coincide, with average losses of 4.7 dB and maximum losses of 5.1 dB for both paths. The *S*_11_, although not as effective as the LO balun with the hybrid impedance transformation scheme, still remains below −7.2 dB. Figure 11b,c show the amplitude–phase imbalance after employing the compensation network and its comparison with the conventional method, respectively. The maximum in-band amplitude error is only 0.05 dB and the phase imbalance ranges from 172° to 187°. Compared to the performance of the structure without compensating measures (4.3 dB, 188°–210°), the RF performances are significantly improved, further demonstrating the effectiveness of the proposed method.

## 4. Isolation-Enhanced DBM Design

**Figure 12:**
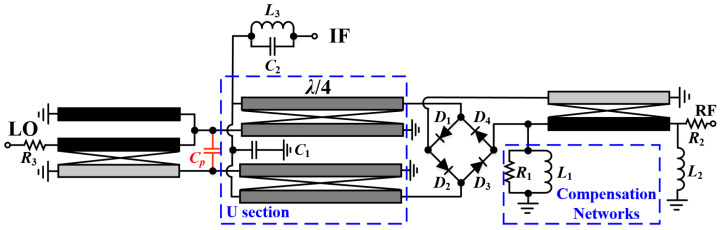
Schematic of the designed 8–30 GHz DBM.

Combined with the proposed broadband Ruthroff-type TLT baluns with excellent differential characteristics, a passive DBM for 8–30 GHz was designed, with the schematic illustrated in Figure 12. The central component of the converter is a ring diode stack, which consists of four source-drain connected transistors, all measuring 2 × 35 µm. The capacitor *C*_1_ serves as an IF filter in addition to functioning as an RF return grounding element. In addition, the inductor *L*_3_ and capacitor *C*_2_ together form a parallel resonant network, which further contributes to the improvement in LO–IF and RF–IF isolation while filtering. The corresponding initial parameters of the whole design are displayed in Table 2 for reference.

**Figure 13:**
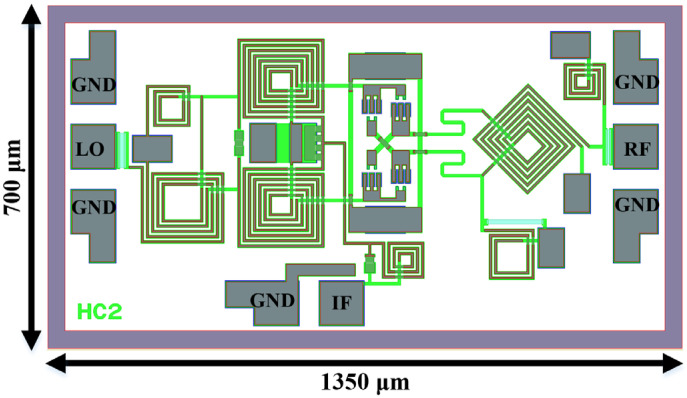
Overall view of the DBM with enhanced isolations.

The passive DBM was designed using 0.15 µm GaAs commercial p-HEMT technology and the corresponding chip overview is presented in Figure 13, with a compact size of 700 µm × 1350 µm, including all on-wafer probing pads. Benefiting from the significant differential characteristics of the proposed baluns, the mixer achieves enhanced port-to-port isolations. All performances are shown below. It is noteworthy that the presented results are based on the method of the “combination of partially measured data and simulation”, where the diode’s performance is measured and the other passive circuits are simulated. The data for diodes are provided by an on-wafer measurement system based on a four-port vector network analyzer, Ceyear 3672E (made by Ceyear, Qingdao, Shandong, China). The above design strategy was adopted primarily for engineering funding reasons, i.e., to approximate the actual situation as accurately as possible at a low cost. In addition, based on previous design experience and several successful commercial chips, this co-simulation methodology nearly matches the performance of the real fabricated chips, and thus the current results are useful for comparison and reference to illustrate the effectiveness of the enhanced circuits.

**Figure 14:**
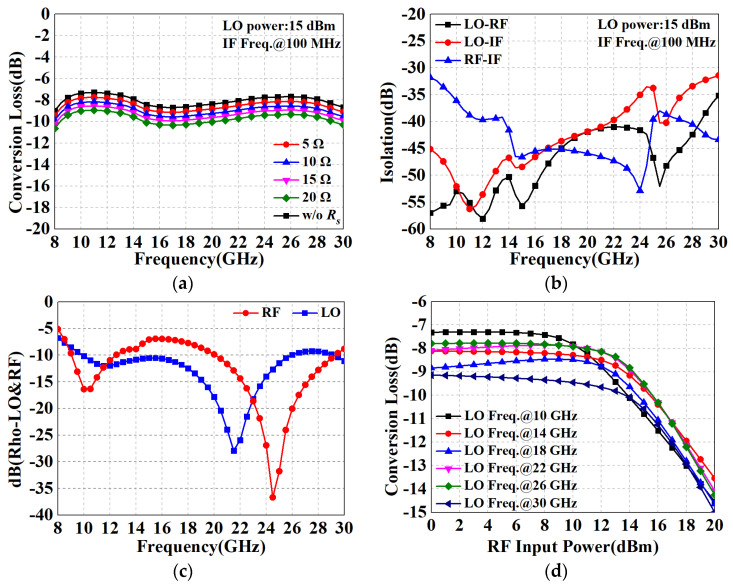
Down-conversion performance results of the designed DBM. (**a**) CL versus RF frequency for various series resistors *R_s_*; (**b**) isolation performances of LO–IF, LO–RF, and RF–IF; (**c**) return losses of the LO port and RF port; (**d**) 1 dB power compression point.

Figure 14 shows the RF performance in down-conversion mode. The conversion loss (CL) with respect to RF frequency for different series resistors *R_s_* is shown in Figure 14a, while the IF frequency and LO power are fixed at 100 MHz and 15 dBm. The designed DBM exhibits a CL of 7.7 dB to 9.2 dB at RF frequency from 8 to 30 GHz, indicating satisfactory flatness while maintaining a relatively low CL. Furthermore, it can be noticed that CL gradually deteriorates at a rate close to −0.1 dB/Ω as *R_s_* increases. However, it must be emphasized that the introduction of *R_s_* improves the port standing wave as well as the linearity of the mixer (i.e., the P_1dB_ metric). Therefore, proper tuning of *R_s_* within a reasonable range can be an effective trade-off between the various RF performances. According to Figure 14b, it features LO-to-RF, LO-to-IF, and RF-to-IF isolations of 36–58 dB, 31–57 dB, and 32–52 dB, respectively. Figure 14c illustrates the matching performance of the LO port and RF port, which are both below −5 dB. Figure 14d shows the CL versus RF power with the IF frequency fixed at 100 MHz and the LO frequency set to be in steps of per 4 GHz in the range of 10–30 GHz. All curves have input 1 dB compression power in the 10–14 dBm range, which implies good linearity.

**Figure 15:**
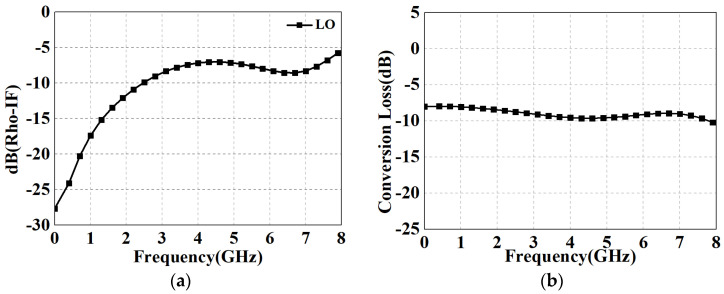
Up-conversion performance results of the designed DBM. (**a**) Return losses of the IF port; (**b**) 3 dB bandwidth at 15 GHz low LO operating frequency.

Figure 15 shows the RF performance in up-conversion mode. Figure 15a shows the return loss of the IF port, which is also below −5 dB, maintaining relatively good matching performance. Based on the results in Figure 15b with the LO operating frequency set to 15 GHz, the 3 dB IF bandwidth of the mixer is up to 8 GHz. Table 3 summarizes the performance of the designed fundamental DBM and it compared with other broadband designs with state-of-the-art methods. It is important to note that although only the data for the diodes are obtained from measurements, this method is highly accurate, which has been verified several times, and thus providing a relatively objective picture of the circuit performance. According to the reference data, the proposed GaAs DBM consumes no DC power and achieves enhanced isolations as well as relatively lower conversion loss with less chip area, not only due to the technology-dependent diode quality as well as conductor losses but also due to the modified Ruthroff-type TLT baluns employed. In fact, we found that the most dominant contributors to the deviation of the measured results from the simulation results are as follows:

(1) When operating at higher frequencies, there is not enough power available to drive the LO port, resulting in a deterioration of the performance.
(2) The impedance of the diode model in the PDK supplied by the foundry deviates from the actual situation, resulting in frequency offset (usually shifted toward the high-frequency band).

Regarding issue-1, the measurement scheme with an external amplifier module can fully cover the operating frequency band (8–30 GHz) required for this design as well as satisfy the driving power requirement (15 dBm), and hence will not introduce too much variation. For issue-2, the measured diode data adopted in this article are used precisely to avoid this problem. In addition, it is necessary to clarify that the design deliberately keeps the spacing between the U section and the spiraled lines at least six times the line width in order to avoid direct coupling between them. Crucially, the U section and the spiraled lines are optimized for performance as a whole during simulation, so the coupling has been taken into account as well. In summary, the comparisons in Table 3 are valuable to illustrate the effectiveness of the circuit.

## 5. Conclusions

For applications in wideband passive DBM design, two modified Ruthroff-type TLT baluns are proposed in this paper, which utilize shunt capacitors and U sections to address the step-down impedance transformation issue within a relatively compact layout. Due to the effective compensation technique, the proposed balun achieves excellent broadband differential characteristics, which further contributes to the improvement in mixer isolation. Compared to other designs of similar frequency bands, the designed DBM achieves relatively low conversion loss, sufficient P_1dB_, and high LO–RF isolation with a compact size, and is therefore ideal for highly integrated and ultra-wideband transceiver systems.

## Figures and Tables

**Table 1:** Comparison of balun performance with other SOTA methods.

**Ref.**	[29]	[30]	[31] ^$^	[16] ^$^	[32] ^$^	This Work ^$^
**RF Freq. (GHz)**	14–28 (66.7%)	7.5–30 (120%)	13–20 (42.4%)	15–60 (120%)	15–65 (125%)	8–30 (116%)
**Technology**	0.15 µm GaAs	0.15 µm GaAs	0.13 µm SiGe	0.15 µm GaAs	0.18 µm CMOS	0.15 µm GaAs
**Topology**	Spiral Marchand balun	Spiral Transformer balun	Spiral Transformer balun	MCL balun	Broadside-coupled balun	Ruthroff-type TLT balun
**Amp. Imb. (dB)**	1	5	<0.7	<1.1	<1	<0.8
**Phase Imb. (deg.)**	±10	<10	<3	<2.5	>5	<4.5
IL (dB) *	1	4.5	1.3	1	5.5	2.2
**Area (mm^2^)**	0.26	0.24 ^#^	N/A	0.72 ^#^	0.23	0.28 ^#^

* Approx. insertion-loss values at midband; ^#^ includes pads; ^$^ simulation results; N/A: not applicable.

**Table 2:** Parameter values, all components.

	*R*_1_	*R*_2_	*R*_3_	*L*_1_	*L*_2_	*L*_3_	*C*_1_	*C*_2_	*D*_1_–*D*_4_
value	631 Ω	3.2 Ω	6.3 Ω	1 nH	0.68 nH	0.7 nH	0.84 pF	0.21 pF	2 × 35 µm

**Table 3:** Performance comparison with other mixers.

**Ref.**	[6]	[16]	[22]	[33]	[34]	This work **^$^**
**RF Freq. (GHz)**	6–20	18–50	5.4–21.8	7–14	14–22	8–30
**Technology**	0.25 µm GaAs pHEMT	0.15 µm GaAs pHEMT	0.25 µm GaAs pHEMT	0.25 µm GaAs HEMT	0.15 µm GaAs pHEMT	0.15 µm GaAs pHEMT
**CL (dB)**	−6–−10	−8.7–−10.8	−4–−7.4	−7–−9 *	−8–−10.5 *	−7.7–−9.2
**LO–RF (dB)**	29–36 *	33–51	>23.5	30–50	30–44	36–58
**LO–IF (dB)**	25–55 *	28–55	>54.7	N/A	28–60	31–57
**RF–IF (dB)**	N/A	32–48	N/A	N/A	20–30	32–52
**P_LO_ (dBm)**	15	15	2	13	13	15
**P_dc_ (mW)**	23	0	0	0	0	0
**Chip area (mm^2^)**	N/A	0.95 × 1.65	2 × 3.2	1 × 1.5	0.9 × 1.5	0.7 × 1.35

* Read from the figure; **^$^** simulation with measured diodes. N/A: not applicable.

## Data Availability

The data that support the findings of this study are available from the author upon reasonable request.
